# 2-Methyl-*N*-phenyl­benzamide

**DOI:** 10.1107/S1600536807068821

**Published:** 2008-01-09

**Authors:** B. Thimme Gowda, Sabine Foro, B. P. Sowmya, Hartmut Fuess

**Affiliations:** a Department of Chemistry, Mangalore University, Mangalagangotri 574 199, Mangalore, India; b Institute of Materials Science, Darmstadt University of Technology, Petersenstrasse 23, D-64287 Darmstadt, Germany

## Abstract

In the structure of the title compound (NP2MBA), C_14_H_13_NO, the conformation of the C—O bond is *syn* to the *ortho*-methyl substituent in the benzoyl phenyl ring, while the N—H bond is *anti* to the *ortho*-methyl substituent. The structure of NP2MBA closely resembles that of 2-chloro-*N*-phenyl­benzamide, with similar bond parameters. The dihedral angle between the phenyl and benzoyl rings is 88.05 (5)°. Mol­ecules are linked into a chain through N—H⋯O hydrogen bonding.

## Related literature

For related literature, see: Gowda *et al.* (2003 ▶, 2007 ▶, 2008 ▶).
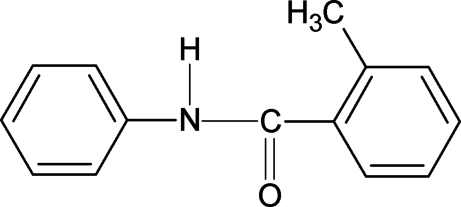


## Experimental

### 

#### Crystal data

C_14_H_13_NO
*M* *_r_* = 211.25
Orthorhombic, 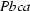

*a* = 14.404 (1) Å
*b* = 8.6824 (6) Å
*c* = 18.710 (1) Å
*V* = 2339.9 (3) Å^3^
*Z* = 8
Mo *K*α radiation
μ = 0.08 mm^−1^
*T* = 100 (2) K
0.40 × 0.20 × 0.16 mm

#### Data collection

Oxford Diffraction Xcalibur diffractometer with Sapphire CCD detector
Absorption correction: multi-scan (*SCALE3 ABSPACK*; Oxford Diffraction, 2007 ▶) *T*_min_ = 0.970, *T*_max_ = 0.981
11005 measured reflections
2387 independent reflections
1686 reflections with *I* > 2σ(*I*)
*R*_int_ = 0.029

#### Refinement

*R*[*F* ^2^ > 2σ(*F* ^2^)] = 0.038
*wR*(*F* ^2^) = 0.115
*S* = 1.05
2387 reflections
149 parameters
H atoms treated by a mixture of independent and constrained refinement
Δρ_max_ = 0.24 e Å^−3^
Δρ_min_ = −0.21 e Å^−3^

### 

Data collection: *CrysAlis CCD* (Oxford Diffraction, 2004 ▶); cell refinement: *CrysAlis RED* (Oxford Diffraction, 2007 ▶); data reduction: *CrysAlis RED*; program(s) used to solve structure: *SHELXS97* (Sheldrick, 2008 ▶); program(s) used to refine structure: *SHELXL97* (Sheldrick, 2008 ▶); molecular graphics: *PLATON* (Spek, 2003 ▶); software used to prepare material for publication: *SHELXS97*.

## Supplementary Material

Crystal structure: contains datablocks I, global. DOI: 10.1107/S1600536807068821/dn2308sup1.cif

Structure factors: contains datablocks I. DOI: 10.1107/S1600536807068821/dn2308Isup2.hkl

Additional supplementary materials:  crystallographic information; 3D view; checkCIF report

## Figures and Tables

**Table 1:** Hydrogen-bond geometry (Å, °)

*D*—H⋯*A*	*D*—H	H⋯*A*	*D*⋯*A*	*D*—H⋯*A*
N1—H1N⋯O1^i^	0.878 (17)	2.012 (18)	2.8751 (16)	167.7 (15)

Symmetry code: (i) 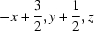.

**Fig. 1.**
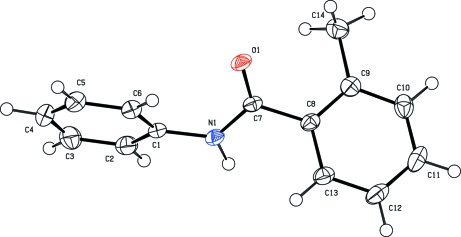
Molecular structure of the title compound, showing the atom labeling scheme. The displacement ellipsoids are drawn at the 50% probability level. H atoms are represented as small spheres of arbitrary radii.

**Fig. 2.**
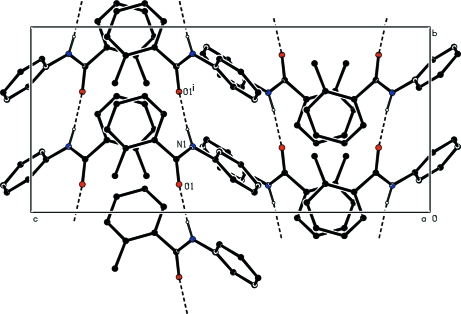
Molecular packing of the title compound with hydrogen bonding shown as dashed lines.H atoms not involved in hydrogen bondings have been omitted for clarity. [Symmetry code: (i) 3/2 - x, 1/2 + y, z]

## supplementary crystallographic information

### Comment

As part of a study of the substituent effects on the structures of benzanilides, in the present work, the structure of 2-methyl-*N*-(phenyl)benzamide (NP2MBA) has been determined (Gowda, *et al.*, 2003; 2007; 2008). In the structure of NP2MBA,(Fig. 1), the conformation of the C—O bond is *syn* to the *ortho*-methyl substituent in the benzoyl phenyl ring, while the N—H bond is anti to the *ortho*-methyl substituent. The bond parameters in NP2MBA are similar to those in 2-chloro-*N*-(phenyl)-benzamide (Gowda, *et al.*,2003), 2-chloro-*N*-(2-chlorophenyl)-benzamide (Gowda, *et al.*,2007), *N*-(4-methylphenyl)-benzamide (Gowda, *et al.*,2008) and other benzanilides. The dihedral angle between the phenyl and benzoyl rings in NP2MBA is 88.05 (5)°. The packing diagram of NP2MBA molecules showing the hydrogen bonds N1—H1N···O1 (Table 1) involved in the formation of molecular chain is shown in Fig. 2.

### Experimental

The title compound was prepared according to the literature method (Gowda *et al.*, 2003). The purity of the compound was checked by determining its melting point. It was characterized by recording its infrared and NMR spectra. Single crystals of the title compound were obtained from an ethanolic solution and used for X-ray diffraction studies at room temperature.

### Refinement

The NH atom was located in difference map with N—H = 0.88 (2) %A. The other H atoms were positioned with idealized geometry using a riding model with C—H = 0.95–0.98 Å A l l H atoms were refined with isotropic displacement parameters (set to 1.2 times of the *U*_eq_ of the parent atom).

### Crystal data

**Table:**

C_14_H_13_NO	*F*_000_ = 896
*M**_r_* = 211.25	*D*_x_ = 1.199 Mg m^−^^3^
Orthorhombic, *P**b**c**a*	Mo *K*α radiation λ = 0.71073 Å
Hall symbol: -P 2ac 2ab	Cell parameters from 3291 reflections
*a* = 14.404 (1) Å	θ = 1.5–26.9º
*b* = 8.6824 (6) Å	µ = 0.08 mm^−^^1^
*c* = 18.710 (1) Å	*T* = 100 (2) K
*V* = 2339.9 (3) Å^3^	Rod, colourless
*Z* = 8	0.40 × 0.20 × 0.16 mm

### Data collection

**Table:**

Oxford Diffraction Xcalibur diffractometer with Sapphire CCD Detector	2387 independent reflections
Radiation source: fine-focus sealed tube	1686 reflections with *I* > 2σ(*I*)
Monochromator: graphite	*R*_int_ = 0.029
*T* = 100(2) K	θ_max_ = 26.4º
Rotation method data acquisition using ω and φ scans.	θ_min_ = 2.6º
Absorption correction: multi-scan(SCALE3 ABSPACK; Oxford Diffraction, 2007)	*h* = −17→17
*T*_min_ = 0.970, *T*_max_ = 0.981	*k* = −10→10
11005 measured reflections	*l* = −23→23

### Refinement

**Table:**

Refinement on *F*^2^	Secondary atom site location: difference Fourier map
Least-squares matrix: full	Hydrogen site location: inferred from neighbouring sites
*R*[*F*^2^ > 2σ(*F*^2^)] = 0.038	H atoms treated by a mixture of independent and constrained refinement
*wR*(*F*^2^) = 0.115	*w* = 1/[σ^2^(*F*_o_^2^) + (0.0589*P*)^2^ + 0.6762*P*] where *P* = (*F*_o_^2^ + 2*F*_c_^2^)/3
*S* = 1.05	(Δ/σ)_max_ = 0.007
2387 reflections	Δρ_max_ = 0.24 e Å^−^^3^
149 parameters	Δρ_min_ = −0.21 e Å^−^^3^
Primary atom site location: structure-invariant direct methods	Extinction correction: none

### Special details

**Table:**

Geometry. All e.s.d.'s (except the e.s.d. in the dihedral angle between two l.s. planes) are estimated using the full covariance matrix. The cell e.s.d.'s are taken into account individually in the estimation of e.s.d.'s in distances, angles and torsion angles; correlations between e.s.d.'s in cell parameters are only used when they are defined by crystal symmetry. An approximate (isotropic) treatment of cell e.s.d.'s is used for estimating e.s.d.'s involving l.s. planes.
Refinement. Refinement of *F*^2^ against ALL reflections. The weighted *R*-factor *wR* and goodness of fit *S* are based on *F*^2^, conventional *R*-factors *R* are based on *F*, with *F* set to zero for negative *F*^2^. The threshold expression of *F*^2^ > σ(*F*^2^) is used only for calculating *R*-factors(gt) *etc*. and is not relevant to the choice of reflections for refinement. *R*-factors based on *F*^2^ are statistically about twice as large as those based on *F*, and *R*- factors based on ALL data will be even larger.

### Fractional atomic coordinates and isotropic or equivalent isotropic displacement parameters (Å^2^)

**Table:**

	*x*	*y*	*z*	*U*_iso_*/*U*_eq_	
C1	0.69394 (10)	0.28490 (16)	0.53783 (8)	0.0226 (3)	
C2	0.60165 (11)	0.32710 (18)	0.52773 (9)	0.0298 (4)	
H2	0.5740	0.4029	0.5575	0.036*	
C3	0.55046 (12)	0.2578 (2)	0.47398 (10)	0.0395 (5)	
H3	0.4876	0.2869	0.4667	0.047*	
C4	0.59039 (14)	0.1463 (2)	0.43075 (10)	0.0421 (5)	
H4	0.5544	0.0966	0.3950	0.051*	
C5	0.68238 (13)	0.1079 (2)	0.43987 (9)	0.0360 (4)	
H5	0.7102	0.0334	0.4094	0.043*	
C6	0.73475 (11)	0.17696 (18)	0.49311 (8)	0.0276 (4)	
H6	0.7983	0.1505	0.4990	0.033*	
C7	0.80898 (9)	0.28653 (16)	0.63583 (7)	0.0197 (3)	
C8	0.85208 (9)	0.38985 (16)	0.69023 (8)	0.0203 (3)	
C9	0.85849 (9)	0.34624 (16)	0.76232 (8)	0.0231 (3)	
C10	0.90322 (10)	0.44704 (19)	0.80890 (9)	0.0284 (4)	
H10	0.9075	0.4210	0.8581	0.034*	
C11	0.94171 (11)	0.58472 (19)	0.78533 (9)	0.0323 (4)	
H11	0.9735	0.6498	0.8180	0.039*	
C12	0.93373 (11)	0.62687 (18)	0.71470 (9)	0.0310 (4)	
H12	0.9590	0.7218	0.6986	0.037*	
C13	0.88876 (10)	0.53029 (16)	0.66721 (9)	0.0248 (4)	
H13	0.8828	0.5597	0.6185	0.030*	
C14	0.81853 (11)	0.19677 (19)	0.78942 (8)	0.0311 (4)	
H14A	0.8571	0.1108	0.7730	0.037*	
H14B	0.7552	0.1842	0.7711	0.037*	
H14C	0.8172	0.1981	0.8418	0.037*	
N1	0.74373 (8)	0.35464 (14)	0.59463 (7)	0.0220 (3)	
H1N	0.7283 (11)	0.448 (2)	0.6078 (9)	0.026*	
O1	0.83366 (7)	0.15084 (11)	0.62885 (6)	0.0245 (3)	

### Atomic displacement parameters (Å^2^)

**Table:**

	*U*^11^	*U*^22^	*U*^33^	*U*^12^	*U*^13^	*U*^23^
C1	0.0295 (8)	0.0138 (7)	0.0244 (7)	−0.0039 (6)	−0.0054 (6)	0.0032 (6)
C2	0.0308 (8)	0.0223 (8)	0.0362 (9)	−0.0009 (7)	−0.0051 (7)	0.0040 (7)
C3	0.0335 (9)	0.0373 (10)	0.0477 (10)	−0.0048 (8)	−0.0176 (8)	0.0095 (9)
C4	0.0563 (12)	0.0291 (9)	0.0410 (10)	−0.0117 (9)	−0.0223 (9)	0.0006 (9)
C5	0.0541 (11)	0.0221 (8)	0.0316 (9)	−0.0039 (8)	−0.0079 (8)	−0.0018 (8)
C6	0.0348 (8)	0.0204 (8)	0.0276 (8)	−0.0022 (7)	−0.0039 (7)	0.0007 (7)
C7	0.0189 (7)	0.0154 (7)	0.0248 (7)	−0.0018 (5)	0.0013 (6)	0.0015 (6)
C8	0.0172 (7)	0.0154 (7)	0.0284 (8)	0.0023 (5)	−0.0008 (6)	−0.0020 (6)
C9	0.0183 (7)	0.0217 (7)	0.0294 (8)	0.0040 (6)	−0.0005 (6)	−0.0019 (7)
C10	0.0249 (8)	0.0316 (9)	0.0287 (8)	0.0065 (7)	−0.0055 (7)	−0.0063 (8)
C11	0.0246 (8)	0.0273 (8)	0.0452 (10)	0.0000 (7)	−0.0082 (7)	−0.0151 (8)
C12	0.0263 (8)	0.0190 (8)	0.0478 (10)	−0.0038 (6)	−0.0030 (7)	−0.0031 (8)
C13	0.0235 (7)	0.0176 (7)	0.0333 (8)	0.0001 (6)	−0.0013 (6)	0.0009 (7)
C14	0.0336 (9)	0.0299 (9)	0.0298 (8)	−0.0022 (7)	−0.0004 (7)	0.0052 (8)
N1	0.0261 (6)	0.0126 (6)	0.0273 (7)	0.0019 (5)	−0.0033 (6)	−0.0017 (6)
O1	0.0274 (5)	0.0124 (5)	0.0336 (6)	0.0014 (4)	−0.0033 (5)	−0.0018 (5)

### Geometric parameters (Å, °)

**Table:**

C1—C6	1.387 (2)	C8—C13	1.397 (2)
C1—C2	1.392 (2)	C8—C9	1.404 (2)
C1—N1	1.4180 (18)	C9—C10	1.393 (2)
C2—C3	1.385 (2)	C9—C14	1.507 (2)
C2—H2	0.9500	C10—C11	1.390 (2)
C3—C4	1.387 (3)	C10—H10	0.9500
C3—H3	0.9500	C11—C12	1.376 (2)
C4—C5	1.377 (3)	C11—H11	0.9500
C4—H4	0.9500	C12—C13	1.383 (2)
C5—C6	1.386 (2)	C12—H12	0.9500
C5—H5	0.9500	C13—H13	0.9500
C6—H6	0.9500	C14—H14A	0.9800
C7—O1	1.2375 (17)	C14—H14B	0.9800
C7—N1	1.3516 (18)	C14—H14C	0.9800
C7—C8	1.492 (2)	N1—H1N	0.878 (17)
			
C6—C1—C2	120.05 (14)	C10—C9—C8	117.51 (14)
C6—C1—N1	121.76 (13)	C10—C9—C14	120.47 (14)
C2—C1—N1	118.19 (13)	C8—C9—C14	122.01 (13)
C3—C2—C1	119.53 (16)	C11—C10—C9	121.78 (15)
C3—C2—H2	120.2	C11—C10—H10	119.1
C1—C2—H2	120.2	C9—C10—H10	119.1
C2—C3—C4	120.41 (16)	C12—C11—C10	120.01 (15)
C2—C3—H3	119.8	C12—C11—H11	120.0
C4—C3—H3	119.8	C10—C11—H11	120.0
C5—C4—C3	119.73 (16)	C11—C12—C13	119.66 (15)
C5—C4—H4	120.1	C11—C12—H12	120.2
C3—C4—H4	120.1	C13—C12—H12	120.2
C4—C5—C6	120.53 (17)	C12—C13—C8	120.56 (15)
C4—C5—H5	119.7	C12—C13—H13	119.7
C6—C5—H5	119.7	C8—C13—H13	119.7
C5—C6—C1	119.69 (15)	C9—C14—H14A	109.5
C5—C6—H6	120.2	C9—C14—H14B	109.5
C1—C6—H6	120.2	H14A—C14—H14B	109.5
O1—C7—N1	123.79 (13)	C9—C14—H14C	109.5
O1—C7—C8	121.65 (12)	H14A—C14—H14C	109.5
N1—C7—C8	114.53 (12)	H14B—C14—H14C	109.5
C13—C8—C9	120.44 (14)	C7—N1—C1	126.32 (12)
C13—C8—C7	118.16 (13)	C7—N1—H1N	114.9 (11)
C9—C8—C7	121.37 (13)	C1—N1—H1N	118.5 (11)
			
C6—C1—C2—C3	1.6 (2)	C13—C8—C9—C14	179.07 (13)
N1—C1—C2—C3	−178.20 (14)	C7—C8—C9—C14	−2.9 (2)
C1—C2—C3—C4	0.5 (3)	C8—C9—C10—C11	−1.1 (2)
C2—C3—C4—C5	−2.1 (3)	C14—C9—C10—C11	179.21 (14)
C3—C4—C5—C6	1.7 (3)	C9—C10—C11—C12	2.0 (2)
C4—C5—C6—C1	0.4 (2)	C10—C11—C12—C13	−1.2 (2)
C2—C1—C6—C5	−2.0 (2)	C11—C12—C13—C8	−0.5 (2)
N1—C1—C6—C5	177.73 (14)	C9—C8—C13—C12	1.5 (2)
O1—C7—C8—C13	126.10 (15)	C7—C8—C13—C12	−176.65 (13)
N1—C7—C8—C13	−52.29 (17)	O1—C7—N1—C1	−0.2 (2)
O1—C7—C8—C9	−51.99 (19)	C8—C7—N1—C1	178.11 (13)
N1—C7—C8—C9	129.62 (14)	C6—C1—N1—C7	−35.5 (2)
C13—C8—C9—C10	−0.7 (2)	C2—C1—N1—C7	144.32 (15)
C7—C8—C9—C10	177.40 (13)		

### Hydrogen-bond geometry (Å, °)

**Table:**

*D*—H···*A*	*D*—H	H···*A*	*D*···*A*	*D*—H···*A*
N1—H1N···O1^i^	0.878 (17)	2.012 (18)	2.8751 (16)	167.7 (15)

Symmetry codes: (i) −*x*+3/2, *y*+1/2, *z*.
